# Prehospital misdiagnosis of acute cerebral disease for acute coronary syndrome: a retrospective study

**DOI:** 10.1186/s13049-022-01063-9

**Published:** 2022-12-23

**Authors:** Josefin Grabert, Ulrich Heister, Andreas Mayr, Andrea Kirfel, Christian Staerk, Tobias Fleckenstein, Markus Velten

**Affiliations:** 1 Department of Anaesthesiology and Intensive Care Medicine, University Hospital Bonn, Venusberg-Campus 1, 53127 Bonn, Germany; 2 Emergency Medical Service Bonn, Bonn, Germany; 3 Department of Medical Biometry, Informatics and Epidemiology, University Hospital Bonn, Bonn, Germany

## Abstract

**Objective:**

In cerebrovascular accidents symptoms, laboratory results and electrocardiogram (ECG) changes can mimic acute coronary syndrome (ACS) and is subsumed as neurogenic stunned myocardium. So far, data regarding the frequency of cerebrovascular accidents misdiagnosed for ACS in a prehospital setting are missing. This study aims to quantify misdiagnoses and discover discriminating features.

**Methods:**

In a retrospective cohort study, prehospital and hospital medical records of all patients treated by physician-staffed emergency medical teams in the city of Bonn (Germany) with suspected ACS in 2018 were evaluated regarding medical history, prehospital symptoms and findings as well as hospital diagnoses.

**Results:**

From 758 patients admitted for presumed ACS, 9 patients (1.2%, 95% CI: 0.5–2.2%) suffered from acute cerebral disease (ACD group). Mainly, diagnoses were cerebrovascular accidents and one case of neuroborreliosis. A history of intracranial haemorrhage was found more often in the ACD group compared to the remaining cohort (OR 19, p = 0.01), while a history of arterial hypertension was less frequent (OR 0.22, p = 0.03). Presentation with headaches (OR 10.1, p = 0.03) or neurological symptoms (OR 16.9, p = 0.01) occurred more frequent in the ACD group. ECG changes were similar between groups.

**Conclusion:**

Acute cerebral disease misdiagnosed for ACS seems more common than assumed. Out of 758 patients with presumed ACS, 9 patients (1.2%) suffered from ACD, which were cerebrovascular accidents mainly. This is highly relevant, since prehospital treatment with heparin and acetylsalicylic acid is indicated in ACS but contraindicated in cerebrovascular accidents without further diagnostics. Thus, discriminating these patients is crucial. An attentive patient history and examination may be the key to differentiating ACD. Due to small ACD group size, further studies are needed.

## Introduction

Cardiovascular diseases are a major cause of morbidity and mortality, responsible for approximately one third of all deaths worldwide with acute coronary syndrome (ACS) and stroke being the most prevalent conditions sharing similar risk factors and pathophysiology [1]. Accordingly, main symptoms such as chest pain, paresis and dysphasia are common reasons for demands in emergency medical services (EMS).

First observations of a connection between acute cerebral disease (ACD) and cardiac dysfunction were made by Cushing in 1903[2]. Later on, these coinciding cerebral and cardiac findings were subsumed as neurogenic stress cardiomyopathy, neurogenic heart syndrome or neurogenic stunned myocardium (NSM)[3–5]. Although detailed mechanisms are not fully understood yet, it is hypothesized that cerebrovascular accidents may present atypically and mimic ACS and with neurally released catecholamine surge being involved[4]. Therefore, patients suffering from cerebrovascular accidents (ischemic or haemorrhagic) may present with chest pain, arrhythmias and ECG changes similar to ACS [5]. Further diagnostics may reveal elevated troponin as well as ventricular wall abnormalities and pulmonary oedema [3, 4, 6].

In prehospital emergency medicine, diagnostic means are limited and emergency physicians have to rely on a thorough medical history and clinical examination. A definite diagnosis of ACS or cerebrovascular accidents depends on laboratory results as well as radiographic findings (i.e., computed tomography, magnetic resonance imaging, angiography). Nevertheless, decisions on prehospital therapy and admission to a suitable hospital are essential for a timely initiation of an adequate therapy. Particularly the loading doses of heparin and acetylsalicylic acid (ASA) are eminent and evident in ACS but may be fatal in cerebrovascular accidents [7].

To our knowledge, prehospital data evaluating the incidence of ACD in suspected ACS are missing. Therefore, this study aims to number misdiagnosed ACD and detect potential discriminating features.

## Methods

### Study design

This retrospective cohort study was performed in accordance with §15 of the Medical Association North Rhine’s professional code of conduct. Due to the retrospective design of this study, an informed consent was waved by the Ethics Committee of the University Hospital Bonn, Germany (No. 054/22).

### Setting

In Germany, the emergency medical services (EMS) include physician-staffed Emergency Medical Teams (PEMT) in addition to ambulances that are dispatched to the scene depending on case severity. In the city of Bonn, the EMS system consists of 17 ambulances and three PEMTs serving approximately 320,000 residents.

Medical records of all patients treated by a PEMT between January 1st 2018 and December 31st 2018 were reviewed and out-of-hospital cases with suspected ACS identified. All patients were diagnosed and treated by an emergency physician according to the EMS Bonn institutional standards as well as international guidelines and transferred to a hospital. Medical records of patients treated by paramedics only were not considered for review, since the complaint of chest pain leads to a PEMT dispatch invariably. Patients who denied or did not require hospital admission were excluded, as were patients under cardiopulmonary resuscitation.

Technical data concerning date, time and location of emergency as well as time of admission and further treating hospital were collected. Medical data including patient age and gender, medical history, current symptoms, heart rate, blood pressure, electrocardiography (ECG) findings and administered drugs were registered. Hospital discharge records were obtained to collect in-hospital tests and validate prehospital diagnoses.

### Statistical analysis

Statistical analysis was performed in the statistical programming environment R. Continuous variables are presented with median, interquartile range (IQR) and range. Categorical variables are displayed as numbers and percentages. Patients were divided into two groups (ACD group vs. remaining cohort) based on the ultimate in-hospital diagnosis. To account for statistical uncertainty in the estimation of ACD incidence based on our cohort, additional to the point estimate also a 95% confidence interval was computed via the method by Clopper and Pearson [8]. To explore the univariate differences between these groups, the non-parametric Wilcoxon-Mann–Whitney U-test was used for continuous variables while for categorical variables, Fisher´s exact test of independence was used. For this exploratory analysis, the two-sided significance level was set to 0.05 without adjusting for multiple testing. Additionally, variables showing relevant differences in these group comparisons were also analysed jointly in a multivariable logistic regression model reporting adjusted Odds-Ratios and corresponding Likelihood-Ratio tests.

## Results

During the evaluated period of one year, 837 patients were treated by a PEMT with presumed ACS and admitted to a hospital. For 79 patients, in-hospital documentation was not available, and therefore they were excluded from further analysis. The remaining 758 patients were composed of 326 females and 432 males. Median age was 72 years, ranging between 18 and 98 years. Forty patients were treated more than once (range 2–4 times) and accounted for 90 cases. All other patients were treated singularly.

**Fig. 1:**
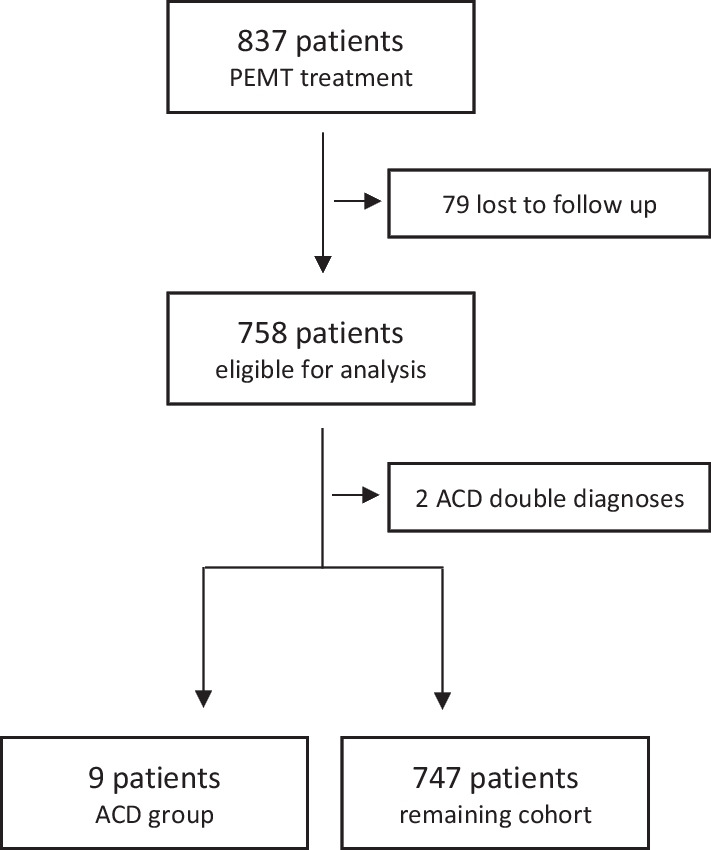
Patient exclusion criteria

Out of the 758 analysed patients admitted for ACS diagnostics and treatment, 11 patients were ultimately diagnosed with ACD (Fig. 1). One patient was excluded from further analysis, because he was diagnosed both with non-ST-elevation myocardial infarction (NSTEMI) and lacunary cerebral infarction. Another patient was excluded, because the exacerbated Parkinson’s disease was presumably caused by underlying sepsis. Thus, 9 (1.2%, CI 0.5–2.2%) out of all admitted patients with presumed ACS suffered from ACD only (ACD group).

Further analysis compared patients in the ACD group to the remaining entity of all admitted patients, regardless of final diagnosis (remaining cohort). Interestingly, based on hospital records, ACS was diagnosed in 283 patients (37.9%) of the remaining cohort only.

Patient baseline characteristics including age and sex were similar between groups and are shown in Table 1. Investigating pre-existing cardiovascular diseases and risk factors, univariate statistical analyses indicated that a history of arterial hypertension was significantly less in the ACD group compared to the remaining cohort (33.3% vs. 68.5%, OR 0.22, CI 0.05–0.86, p = 0.03). Interestingly, systolic blood pressure on presentation was not different between groups (p = 0.8). A history of ischemic heart disease or atrial fibrillation was not significantly different. Chest pain on presentation and amount of antiaggregant agents were distributed similarly between groups.

**Table 1:** Patient baseline characteristics

	ACD group (n = 9)	Remaining cohort (n = 747)	p-value	Missings
*Gender*			p = 0.51	0
Male	4	426		
Female	5	321		
Age [years] (median, IQR, range)	71.9 (23.5; 48.9–79.9)	75.4 (18.6; 18.9–98.9)	p = 0.73	0
*Pre-existing cardiovascular conditions*				
Ischemic heart disease	2	308	p = 0.32	6
Atrial fibrillation	3	143	p = 0.39	6
arterial hypertension	3	512	*p* = *0.03*	*6*
*Pre-existing neurologic conditions*				
Ischemic stroke/TIA	1	71	p = 1	61
Intracranial haemorrhage	2	10	*p* = *0.01*	61
Cerebrovascular disease	0	25	p = 1	61
Number of anticoagulant agents [median, IQR, range]	1.0 (1.0; 0–2)	1.0 (1.0; 0–3)	p = 0.92	44

*ACD* Acute cerebral disease—*TIA* transitory ischemic attack

Final diagnoses in the ACD group were mainly cerebrovascular accidents (Table 2). Two patients were diagnosed with a transitory ischemic attack (TIA), one with TIA of the anterior spinal artery combined with a panic disorder, and one with TIA combined with vertebral artery dissection. Two patients suffered from ischemic stroke. Aneurysmatic subarachnoid haemorrhage (SAH) was present in one patient, traumatic SAH combined with subdural haematoma in another. One patient was diagnosed with neuroborreliosis.

**Table 2:** Patient details of ACD group

No	Symptoms (cardiac)	Symptoms (neurologic)	ECG	GCS	Cardiac history	CNS history	OAC (no)	Final diagnosis
231	Chest pain	headache	ST-E, ST-D	15	aHT	SAH	0	SAH
385	Chest pain	–	–	15	AF	–	1	Neuroborreliosis
469	Epigastric/thoracic pain	–	inverted T	15	AF	ICH	1	tSAH, SDH
485	Chest pain	Hemiparesis, diplopia, vertigo	peaked T	14	CAD, aHT	facial paresis	2	spinal TIA/panic disorder
611	Chest pain	–	–	15	–	–	0	TIA
710	Chest pain	–	ST-E	15	CAD	stroke	1	TIA
741	Unwell	Headache, dysphasia, paraesthesia	ST-D	14	aHT, DM	–	0	lacunar ischemia
782	Chest pain	–	–	15	AF, PE	–	1	MCA ischemia
806	Unwell	syncope	ST-E	15	–	–	0	VA dissection, TIA

*AF* atrial fibrillation, *aHT* arterial hypertension, *CAD* coronary artery disease, *CNS* central nervous system, *DM* diabetes mellitus, *ECG* electrocardiogram, *GCS* Glasgow Coma Scale, *ICH* intracranial haemorrhage, *MCA* medial cerebral artery, *OAC* oral anticoagulants (pre-existing), *PE* pulmonary embolism, *SAH* subarachnoid haemorrhage, *SDH* subdural haematoma, *ST-D* ST-segment depression, *ST-E* ST-segment elevation, *TIA *transitory ischemic attack, *tSAH* traumatic SAH, *VA* vertebral artery

Analysing neurological conditions, only a previous ICH showed significant difference between groups (22.2% in ACD group vs. 1.3% in remaining cohort, OR 19, CI 2.66–92.95, p = 0.01). Statistical analysis indicated no significant difference regarding history of cerebrovascular accidents or carotid artery disease between groups. Clinical presentation with neurological symptoms (paresis, paraesthesia, diplopia or dysphasia) occurred significantly more frequent in the ACD group (22% vs. 1.6%, OR 16.9, CI 2.39–80.52, p = 0.01). The incidence of headaches was significantly higher in the ACD group (22.2% vs. 2.7%, OR 10.1, CI 1.46–45.43, p = 0.03). Glasgow Coma Scale (GCS) on presentation was more often reduced in the ACD group compared to the remaining cohort with 22.2% of patients showing a value < 15 compared to 1.0% in the remaining cohort (OR 9.22, CI 1.33—41.36, p = 0.03). Details on prehospital presentation and treatment are shown in Table 3.

**Table 3:** Prehospital patient characteristics

	ACD group (n = 9)	Remaining cohort (n = 747)	p-value	Missings
*Symptoms on presentation*				
Chest pain	6	598	p = 0.38	9
HEADACHE	2	20	*p* = *0.03*	*14*
Neurological deficit	2	12	*p* = *0.01*	*14*
*Findings on presentation*				
GCS < 15	2	7	*p* = *0.03*	42
Systolic blood pressure [mmHg, median, IQR, range]	150.0 (43.0; 105—210)	166.0 (63.0; 60—240)	p = 0.77	2
ST-elevation on ECG	3	147	p = 0.45	131
ST-depression on ECG	2	158	p = 1	131
*Prehospital treatment*				
Heparin	5	517	p = 0.47	0
ASA	5	496	p = 0.50	4
Nitroglycerin	2	228	p = 0.73	1

GCS Glasgow Coma Scale—*ECG* electrocardiogram—*ASA* Acetylsalicylic acid

Statistical analysis did not indicate differences in ECG findings, i.e., ST-segment deviations or T wave changes. Medication administered out of hospital (ASA, heparin, urapidil, nitroglycerin) and in-hospital troponin values were similar between groups.

Multivariable regression analysis involving four variables showed a significant effect for arterial hypertension (adj. OR 0.137, CI 0.023–0.038, p = 0.01), intracranial haemorrhage (adj. OR 28.6, CI 3.4–189.2, p < 0.01) and neurological deficits (adj. OR 26.7, CI 3.3–165.7, p < 0.01) as well as a borderline effect for headache (adj. OR 7.7, CI 0.98–40.8, p = 0.05).

## Discussion

During an observational period of one year, out of 758 patients treated by a PEMT for ACS symptoms at the EMS Bonn (Germany), 9 patients (1.2%, CI 0.5–2.2%) were characterized by simultaneous chest pain and misinterpreted neurological deficits upon initial presentation and ultimately diagnosed with an ACD mimicking an ACS. To our knowledge, this is the first time the incidence of misdiagnosed ACD for suspected ACS has been evaluated in the prehospital setting and our reported incidence is higher compared to previous reports of the “stroke chameleon” in the clinical setting [9]. Considering that neurological disorders account for approximately 7% of all emergency cases in a representative German study [10], 1.2% misdiagnosed patients seem a relevant amount. Within the herein evaluated EMS, this would result in one misdiagnosed patient every six weeks.

ACS and ACD seem different in pathogenesis and clinical presentation. As observed by Cushing, both share common features such as chest pain, elevated troponin and ECG changes [2]. These symptoms and clinical manifestations, subsumed as neurogenic stunned myocardium (NSM) or stroke chameleon, may explain why, sporadically, suspected ACS proves to be an ACD. We present a retrospective analysis evaluating the quantity of ACD within suspected ACS in a prehospital setting, and potentially discriminating features.

Correct out of hospital discrimination between ACS and ACD is important, because prehospital treatment differs relevantly, hospital allocation depends on prehospital diagnosis using current symptoms as well as medical history, and misallocation may delay time sensitive therapy. For suspected ACS, prehospital treatment includes antiaggregant therapy composed of ASA and heparin dosages [7]. For suspected cerebrovascular accidents, prehospital antiaggregant therapy is contraindicated: in case of haemorrhage per definition, in case of ischemia depending on the time of event and risk of secondary bleeding, defined by computed tomography (CT). In the present cohort, 5 patients in the ACD group (55.6%) received ASA and heparin boluses for suspected ACS. Two of these patients were diagnosed with ICH, 3 suffered from cerebrovascular accidents. Thus, the ICH patients were not only treated inadequately, but received contraindicated medication and were exposed to neurological deterioration. Fortunately, both patients did not develop further neurological deficits as compared to the time of hospital admission.

Out of hospital cardiac arrests (OHCA) were excluded from analysis. OHCA due to cerebrovascular accidents or ACS may mimic each other, but prehospital therapy does not differ, since ERC guidelines do not recommend thrombolytic drugs in suspected OHCA due to ACS [11]. Noteworthy, from a retrospective analysis comparing OHCA from SAH to OHCA from ACS, asystole or pulseless electrical activity seems to be a discriminating characteristic for OHCA due to SAH, especially in the absence of ST-segment deviations [12].

Available data on the link between ACS and ACD refer usually to cerebrovascular accidents [5, 13, 14]. It is proposed, that insular involvement leads to autonomic dysregulation resulting in a catecholamine release, thus leading to myocardial dysfunction with wall movement abnormalities, ECG changes and elevated troponin levels [4]. Consistently, final diagnoses in the ACD group were TIA and ischemic stroke in 5 patients and ICH in 2 patients. Interestingly, one patient suffered from neuroborreliosis and one had a suspected combination of anterior spinal artery TIA and panic attack. Spinal ischemia and myelitis, which may involve the sympathetic nervous system, seem conceivable and have been described in two case reports previously [15, 16]. Sole emotional stress may induce NSM, too, with regard to its previous term of “broken heart syndrome” [17]. Neuroborreliosis as trigger for NSM has not been described, yet. Since neuroborreliosis mainly manifests in neuritis and infectious causes for NSM are rare, its causal relation remains unclear, but NSM induced by limbic encephalitis has been described [18].

Discriminating features for ACD in this population are a history of ICH (OR 19, p = 0.01), headaches on presentation (OR 10.1, p = 0.03) or general appearance of neurological symptoms (OR 16.9, p = 0.01). Although seemingly obvious, correct diagnosis was probably confounded by co-existence of chest pain and ECG changes. This is in concordance with statistical analysis, that revealed no difference between groups regarding chest pain and ECG changes. Statistically, GCS was more often reduced in ACD (2 vs. 7 cases with GCS < 15, p = 0.03), advocating its value as indicator for neurological disorders.

A definite and certain distinction between ACS and ACD cannot be accomplished in a prehospital setting, since diagnoses rely on laboratory and imaging results. The use of point of care troponin tests might be helpful in discriminating ACS, but false negative tests must be considered due to delayed increase of serum troponin levels after onset of symptoms. More importantly, elevated troponin levels are well described in ACD patients. [4, 5] Thus, point of care troponin tests will not aid in discriminating ACS from ACD patients prehospitally. Prehospital echocardiography is not established within the EMS Bonn, but is evolving generally, and might be a helpful tool for the experienced emergency physician. Again, ventricular wall motion abnormalities have been described in ACD patients, not allowing a certain differentiation between ACS and ACD in the prehospital setting. [19] Obviously, cerebral imaging to verify or exclude ACD diagnosis is not available. Given these aspects, emergency physicians have to rely and decide on a focused patient history and careful examination. From the presented cohort, appearance of neurological symptoms or reduced GCS might be the “red flags” in discriminating ACD from ACS and should be considered carefully.

From a retrospective analysis, Bulsara et al. proposed criteria to differentiate ACS from NSM in a clinical setting: missing history of heart disease, new onset of cardiac dysfunction defined as ejection fraction (EF) < 40%, wall motion abnormalities discordant to ECG changes and troponin < 2.8 ng/ml in patients with EF < 40% [19]. In the present cohort, only a missing history of arterial hypertension in the ACD group (OR 0.22, p = 0.03) was differentiating. Since arterial hypertension is a common risk factor for heart disease, this is consistent with Bulsara’s NSM criteria. In contrast, from a retrospective multicenter registry study on clinical differences between ACS and stroke, arterial hypertension was more prevalent in stroke patients. Subgroup analysis of female patients revealed no difference in history of arterial hypertension between ACS and stroke patients [20]. It must be considered, that in the presented study, ACD was not compared to the correctly diagnosed ACS patients, but to suspected ACS patients, which might explain these seemingly contradictory results. Also, a subgroup analysis of female patients was not performed, since data, especially regarding ACD group, was underpowered.

Recent investigations on ICH concomitant to ACS reveal its rareness (0.2–0.4%) but also point out the poorer overall outcome [21, 22]. In both studies patients with concomitant ICH received more antiaggregant drugs. It may be speculated, that ICH was a complication due to therapy for ACS as opposed to the presented cases with suspected NSM. Final certainty on what is cause and what is effect may never be reached, though.

## Limitations

Owing the retrospective design of this analysis, not all data sets are complete. Notably, prehospital ECG diagnoses are missing in a relevant amount (131/758) of data sets. Despite a comprehensive study cohort, results of the exploratory statistical inference must be interpreted with care since the comparative group (ACD group) is very small. This also reflects in the broad confidence intervals obtained.

## Conclusions

This is, to our knowledge, the first study evaluating the incidence of missed ACD in patients suspected for ACS in the prehospital setting. In 1.2% of the reviewed cases, an ACD mimicked an ACS on presentation. Prehospital diagnostic possibilities are limited to patient history and clinical examination, and physicians are bound to consider even slight but important neurological symptoms or uncommon presentations of cerebrovascular accidents. From our data, in particular neurological symptoms or impairment including headaches and a history of ICH are suspicious for ACD. Evaluating these “red flags” might aid the decision to one of two diverging therapeutic strategies, avoid misdiagnoses of acute neurological events and prevent delayed or inadequate therapy.

## Data Availability

The datasets used and/or analysed during the current study are available from the corresponding author on reasonable request.

## Abbreviations

ACD: Acute cerebral disease
ACS: Acute coronary syndrome
AF: Atrial fibrillation
aHT: Arterial hypertension
ASA: Acetylsalicylic acid
CAD: Coronary artery disease
CNS: Central nervous system
CT: Computed tomography
DM: Diabetes mellitus
ECG: Electrocardiogram
EF: Ejection fraction
EMS: Emergency medical service
GCS: Glasgow coma scale
ICH: Intracranial haemorrhage
IQR: Interquartile range
MCA: Medial cerebral artery
NSM: Neurogenic stunned myocardium
NSTEMI: Non-ST elevation myocardial infarction
OAC: Oral anticoagulant
OHCA: Out-of-hospital cardiac arrest
PE: Pulmonary embolism
PEMT: Physician staffed emergency medical team
SAH: Subarachnoid haemorrhage
SDH: Subdural haematoma
ST-D: ST-segment depression
ST-E: ST-segment elevation
TIA: Transitory ischemic attack
tSAH: Traumatic subarachnoid haemorrhage
VA: Vertebral artery
